# ChatGPT, Gemini, and Claude in clinical and dermoscopic image analysis of basal cell carcinoma and its common mimickers: A comparative performance analysis

**DOI:** 10.1016/j.xjidi.2026.100463

**Published:** 2026-02-28

**Authors:** Mehdi Boostani, Christos C. Zouboulis, Giovanni Pellacani, Cristian Navarrete-Dechent, Lucas Boussingault, Tara Kiss, Noah Goldfarb, Carmen Cantisani, Nóra Nádudvari, András Bánvölgyi, Norbert M. Wikonkál, Mariano Suppa, Gyorgy Paragh, Norbert Kiss

**Affiliations:** 1 Department of Dermatology, Roswell Park Comprehensive Cancer Center, Buffalo, New York, USA; 2 Departments of Dermatology, Venereology, Allergology and Immunology, Staedtisches Klinikum Dessau, Brandenburg Medical School Theodor Fontane and Faculty of Health Sciences Brandenburg, Dessau, Germany; 3 Department of Dermatology, Sapienza University of Rome, Rome, Italy; 4 Department of Dermatology, Escuela de Medicina, Pontificia Universidad Catolica de Chile, Santiago, Chile; 5 Department of Dermatology, Hôpital Erasme, HUB, Université Libre de Bruxelles, Brussels, Belgium; 6 Department of Dermato-oncology, Institut Jules Bordet, HUB, Université Libre de Bruxelles, Brussels, Belgium; 7 Department of Dermatology, Venereology and Dermatooncology, Semmelweis University, Budapest, Hungary; 8 Department of Medicine, Medical School, University of Minnesota, Minneapolis, Minnesota, USA; 9 Department of Dermatology, Medical School, University of Minnesota, Minneapolis, Minnesota, USA; 10 Department of Medicine, Minneapolis VA Health Care System, Minneapolis, Minnesota, USA; 11 Department of Dermatology, Minneapolis VA Health Care System, Minneapolis, Minnesota, USA; 12 Central Hospital of Northern Pest–Military Hospital, Budapest, Hungary; 13 Groupe d'Imagerie Cutanée Non-Invasive (GICNI) of the Société Française de Dermatologie (SFD), Paris, France

**Keywords:** Artificial intelligence, Basal cell carcinoma, ChatGPT, Gemini, Large language model

## Abstract

Basal cell carcinoma (BCC) is the most common skin cancer. Off-the-shelf multimodal large language models are widely accessible, yet their performance for BCC remains unclear. The aim of this study was to assess BCC detection (BCC vs non-BCC) and BCC subtype classification from clinical and dermoscopic images using 3 web-based large language models (ChatGPT-5, Gemini 2.5 Flash, Claude Sonnet 4). We evaluated 772 images: 402 from 290 histopathology-confirmed BCCs (290 clinical, 112 dermoscopic) and 370 from an independent BCC-mimicker cohort (250 clinical, 120 dermoscopic). Standardized prompts were used. Primary outcome was BCC detection accuracy; secondary outcomes were subtype-classification accuracy and performance by lesion features. For clinical images, ChatGPT-5 achieved the highest detection accuracy (75%), followed by Claude (64.3%) and Gemini (50.7%). For dermoscopy, Claude performed best (69.8%), compared with ChatGPT-5 (55.2%) and Gemini (50.9%). Accuracy was lower in crusted and flat lesions and higher in exophytic lesions; pigmentation effects were model dependent. Subtype-classification accuracy was modest across models. Images were primarily from European centers with limited skin-type diversity; several subgroups were small. Current web-based large language models are not clinically suitable for BCC detection or subtyping. Dermatology-specific training, transparent reporting, and rigorous prospective validation are required before any clinical use.

## Introduction

Basal cell carcinoma (BCC) is the most common skin cancer and the most frequent malignancy in fair-skinned populations (K et al, 2013). It comprises multiple histopathological subtypes, including superficial BCC (sBCC), nodular BCC (nBCC), infiltrative BCC, micronodular BCC (mnBCC), morpheaform BCC, basosquamous BCC, and others, each differing in biological behavior, prognosis, and recurrence risk (Saldanha et al, 2003; Schmults et al, 2023; Scrivener et al, 2002; Vantuchova and Curik, 2006). Accurate identification is critical for distinguishing BCC from clinical mimickers such as squamous cell carcinoma, melanoma, nevi, adnexal tumors, sebaceous gland hyperplasia, and seborrheic keratosis, among others (Karampinis et al, 2024). Misidentification can lead to overtreatment of benign lesions or delayed diagnosis of potentially aggressive BCCs, underscoring the need for accessible diagnostic tools. Accurate subtyping is also critical because aggressive subtypes such as infiltrative BCC, mnBCC, morpheaform BCC, and basosquamous BCC often require wider surgical margins or Mohs micrographic surgery, whereas less aggressive forms such as sBCC and nBCC may be managed with more conservative approaches (Algarin et al, 2023; Clark et al, 2014; Collier et al, 2018; Firnhaber, 2012; van Kester et al, 2019; Zouboulis et al, 1994).

Currently, BCC diagnosis and subtyping rely mainly on clinical examination; noninvasive skin imaging modalities (ie, dermatoscopy, reflectance confocal microscopy); and, ultimately, the gold standard histopathology (Boone et al, 2016; Boostani et al, 2025a, 2025c, 2025d; Kiss et al, 2019; Kyrgidis et al, 2010; Raasch et al, 2006; Scrivener et al, 2002). Recently, artificial intelligence (AI), particularly large language models (LLMs), has shown promise in diagnosing various dermatologic conditions (Boostani et al, 2025b; Du-Harpur et al, 2020; Hogarty et al, 2020; Paganelli et al, 2024; Salinas et al, 2024; Zhou et al, 2024). Although LLMs were initially used mainly for natural language processing (ie, text only) (Paganelli et al, 2024), modern LLMs now support multimodal inputs, including image interpretation. However, their utility in diagnosing and subtyping BCC, despite its prevalence, remains untested.

Given the widespread availability and publicity of LLMs, individual practitioners and even patients may attempt to use off-the-shelf models for triage or provisional diagnosis. Because such models are not dermatology trained, prospectively validated, or clinically regulated for BCC care, evaluating their real-world performance is essential. The aim of the study was to assess the performance of off-the-shelf multimodal LLMs for 2 tasks: (i) BCC detection (BCC vs non-BCC) and (ii) BCC subtype classification from both clinical and dermoscopic images. The primary outcome was diagnostic performance for BCC detection (sensitivity, specificity, positive predictive value [PPV], negative predictive value [NPV], and accuracy). Secondary/exploratory outcomes included subtype-classification performance overall and by histopathologic subtype and lesion characteristics (pigmentation, crusting, growth pattern). Accordingly, we evaluated AI-driven, web-based multimodal LLMs using clinical and dermoscopic images for both BCC detection and subtype classification.

## Results

In total, we analyzed 772 images: 402 from 290 histopathologically confirmed BCC lesions in 267 consecutive patients (290 clinical, 112 dermoscopic) and 370 from an independent BCC-mimicker cohort (250 clinical, 120 dermoscopic) from 250 lesions that could mimic BCC in 250 patients. The mean number of lesions per patient in the BCC cohort was 1.1 ± 0.3. Detailed demographics and lesion characteristics of the BCC cohort are provided in Table 1. The mean number of lesions per patient in the BCC-mimicker cohort was 1. Detailed demographics and lesion characteristics of the BCC-mimicker cohort are provided in Table 2.

**Table 1:** Patient and Lesion Characteristics in the Basal Cell Carcinoma Cohort

Category	Patient/Lesion Data
Patient characteristics	Total (n = 267)
Age, y	
Mean	71.8 ± 12.3
Median	74
Range	34–98
Sex	
Male	46.8% (125)
Female	53.2% (142)
Fitzpatrick skin type	
Type I	0.7% (2)
Type II	74.5% (199)
Type III	22.8% (61)
Type IV	2% (5)
Lesion characteristics	Total (n = 290)
Lesion distribution	
Head and neck	73.1% (212)
Trunk	19.3% (56)
Extremity	7.6% (22)
Lesion subtype	
Nodular	51% (148)
Superficial	6.6% (19)
Infiltrative	14.8% (43)
Micronodular	7.2% (21)
Other	9.7% (28)
Mixed subtype	10.7% (31)
Ulceration	
Nonulcerated	85.5% (248)
Ulcerated	14.5% (42)
Pigmentation	
Nonpigmented	87.6% (254)
Pigmented	12.4% (36)
Surface	
Noncrusted	77.9% (226)
Crusted	22.1% (64)
Growth pattern	
Flat	89.3% (259)
Exophytic	10.7% (31)

The table shows the descriptive demographics of the enrolled patients and descriptive characteristics of the enrolled basal cell carcinoma lesions included in the study.

**Table 2:** Patient and Lesion Characteristics in the Basal Cell Carcinoma-Mimicker Cohort

Category	Patient/Lesion Data
Patient characteristics	Total (n = 250)
Age, y	
Mean	53.7 ± 18.1
Median	54
Range	19–83
Sex	
Male	47.6% (119)
Female	52.4% (131)
Fitzpatrick skin type	
Type I	6.4% (16)
Type II	43.6% (109)
Type III	35.2% (88)
Type IV	14.8% (37)
Lesion characteristics	Total (n = 250)
Lesion distribution	
Head and neck	47.6% (119)
Extremity	32.8% (82)
Trunk	19.6% (49)
Lesion type	
Squamous cell carcinoma	24% (60)
Naevus	24% (60)
Melanoma	24% (60)
Seborrheic keratosis	12% (30)
Dermatofibromas	4% (10)
Neurofibroma	4% (10)
Actinic keratosis	4% (10)
Keratoacanthomas	4% (10)

The table presents the descriptive demographics of the enrolled patients and descriptive characteristics of the enrolled basal cell carcinoma-mimicker lesions included in the study.

### Accuracy in diagnosing BCC

#### Clinical images

Across all clinical images, the 3 evaluated LLMs showed differing recall (sensitivity) for correctly identifying BCCs (chi-square = 96.46, *P* < .0001) against the histopathologic gold standard. ChatGPT-5 correctly identified 63.4% of BCC cases, followed by Claude Sonnet 4 at 46.6% and Gemini 2.5 Flash at 23.1%. Detailed diagnostic metrics, including diagnostic accuracy, can be found in Table 3.

**Table 3:** Diagnostic Performance of 3 Web-Based LLMs on Clinical Images, Stratified by Lesion Characteristics

Model	Category	Sensitivity	Specificity	PPV	NPV	LR+	LR−	Accuracy
ChatGPT-5	Pigmented	52.8% (CI95: 37–68%)	88.4% (CI95: 83.8–91.8%)	39.6% (CI95: 27–53.7%)	92.9% (CI95: 88.9–95.5%)	4.55 (CI95: 2.87–7.21)	0.53 (CI95: 0.38–0.76)	83.9% (CI95: 79.2–87.7%)
	Nonpigmented	65% (CI95: 58.9–70.6%)	88.4% (CI95: 83.8–91.8%)	85.1% (CI95: 79.4–89.4%)	71.3% (CI95: 66–76%)	5.6 (CI95: 3.93–7.98)	0.4 (CI95: 0.33–0.47)	76.6% (CI95: 72.7–80.1%)
	Crusted	35.9% (CI95: 25.3–48.2%)	88.4% (CI95: 83.8–91.8%)	44.2% (CI95: 31.6–57.7%)	84.4% (CI95: 79.5–88.3%)	3.1 (CI95: 1.93–4.97)	0.72 (CI95: 0.60–0.88)	77.7% (CI95: 72.8–82%)
	Noncrusted	81.4% (CI95: 75.8–85.9%)	88.4% (CI95: 83.8–91.8%)	86.4% (CI95: 81.1%–90.4%)	84% (CI95: 79.1–88%)	7.02 (CI95: 4.96–9.94)	0.21 (CI95: 0.16–0.28)	85.1% (CI95: 81.6–88%)
	Exophytic	80.6% (CI95: 63.7–90.8%)	88.4% (CI95: 83.8–91.8%)	46.3% (CI95: 33.7–59.4%)	97.4% (CI95: 94.4–98.8%)	6.95 (CI95: 4.74–10.2)	0.22 (CI95: 0.11–0.45)	87.5% (CI95: 83.2–90.9%)
	Non-exophytic	61.4% (CI95: 55.3–67.1%)	88.4% (CI95: 83.8–91.8%)	84.6% (CI95: 78.7–89%)	68.8% (CI95: 63.6–73.7%)	5.29 (CI95: 3.71–7.55)	0.44 (CI95: 0.37–0.51)	74.7% (CI95: 70.7–78.2%)
	Overall	63.4% (CI95: 57.8–68.8%)	88.4% (CI95: 83.8–91.8%)	86.4% (CI95: 81.1–90.4%)	67.6% (CI95: 62.3–72.4%)	5.47 (CI95: 3.84–7.79)	0.41 (CI95: 0.35–0.48)	75% (CI95: 71.2–78.5%)
Gemini 2.5 Flash	Pigmented	13.9% (6.1–28.7%)	82.8% (CI95: 77.6–87%)	10.4% (CI95: 4.5–22.2%)	87% (CI95: 82.1–90.7%)	0.81 (CI95: 0.34–1.90)	1.04 (CI95: 0.90–1.20)	74.1% (CI95: 68.8–78.9%)
	Nonpigmented	24.4% (19.5–30%)	82.8% (CI95: 77.6–87%)	59% (CI95: 49.5–68%)	51.9% (CI95: 47–56.7%)	1.42 (CI95: 1.00–2.01)	0.91 (CI95: 0.83–1.00)	53.4% (CI95: 49–57.7%)
	Crusted	14.1% (CI95: 7.6–24.6%)	82.8% (CI95: 77.6–87%)	17.3% (CI95: 9.4–29.7%)	79% (CI95: 73.7–83.5%)	0.82 (CI95: 0.42–1.59)	1.04 (CI95: 0.93–1.16)	68.8% (CI95: 63.5–73.7%)
	Noncrusted	29.6% (CI95: 24.1–35.9%)	82.8% (CI95: 77.6–87%)	60.9% (CI95: 51.6–69.5%)	56.6% (CI95: 51.4–61.5%)	1.72 (CI95: 1.23–2.42)	0.85 (CI95: 0.77–0.94)	57.6% (CI95: 53.1–61.9%)
	Exophytic	29% (CI95: 16.1–46.6%)	82.8% (CI95: 77.6–87%)	17.3% (CI95: 9.4–29.7%)	90.4% (CI95: 85.9–93.6%)	1.69 (CI95: 0.91–3.12)	0.86 (CI95: 0.68–1.08)	76.9% (CI95: 71.6–81.4%)
	Nonexophytic	22.4 (CI95: 17.7–27.9%)	82.8 (CI95: 77.6–87%)	57.4% (CI95: 47.7–66.6%)	50.7% (CI95: 45.9–55.6%)	1.3 (CI95: 0.91–1.86)	0.94 (CI95: 0.86–1.02)	52.1% (CI95: 47.7%–56.4%)
	Overall	23.1% (CI95: 18.6–28.3%)	82.8% (CI95: 77.6–87%)	60.9% (CI95: 51.6–69.5%)	48.1% (CI95: 43.5–52.9%)	1.34 (CI95: 0.95–1.89)	0.93 (CI95: 0.85–1.01)	50.7% (CI95: 46.5–54.9%)
Claude Sonnet 4	Pigmented	58.3% (CI95: 42.2–72.9%)	84.8% (CI95: 79.8–88.7%)	35.6% (CI95: 24.6–48.3%)	93.4% (CI95: 89.4–96%)	3.84 (CI95: 2.57–5.74)	0.49 (CI95: 0.33–0.73)	81.5% (CI95: 76.6–85.5%)
	Nonpigmented	52% (CI95: 45.8–58%)	84.8% (CI95: 79.8–88.7%)	77.6% (CI95: 70.8–83.3%)	63.5% (CI95: 58.2–68.5%)	3.42 (CI95: 2.49–4.69)	0.57 (CI95: 0.49–0.65)	68.3% (CI95: 64.1–72.2%)
	Crusted	37.5% (CI95: 26.7–49.7%)	84.8% (CI95: 79.8–88.7%)	38.7% (CI95: 27.6–51.2%)	84.1% (CI95: 79.1–88.1%)	2.47 (CI95: 1.6–3.8)	0.74 (CI95: 0.61–0.9)	75.2% (CI95: 70.1–79.6%)
	Noncrusted	67.7% (CI95: 61.4–73.5%)	84.8% (CI95: 79.8–88.7%)	80.1% (CI95: 73.9–85.1%)	74.4% (CI95: 69–79.1%)	4.45 (CI95: 3.28–6.05)	0.38 (CI95: 0.31–0.46)	76.7% (CI95: 72.7–80.3%)
	Exophytic	77.4% (CI95: 60.2–88.6%)	84.8% (CI95: 79.8–88.7%)	38.7% (CI95: 27.6–51.2%)	96.8% (CI95: 93.6–98.4%)	5.09 (CI95: 3.59–7.22)	0.27 (CI95: 0.14–0.51)	84% (CI95: 79.2–87.8%)
	Nonexophytic	49.8% (CI95: 43.8–55.9%)	84.8% (CI95: 79.8–88.7%)	77.2% (CI95: 70.3–82.9%)	62% (CI95: 56.7–67%)	3.28 (CI95: 2.39–4.5)	0.59 (CI95: 0.52–0.68)	67% (CI95: 62.8–70.9%)
	Overall	46.6% (CI95: 40.9–52.3%)	84.8% (CI95: 79.8–88.7%)	78% (CI95: 71.3–83.6%)	57.8% (CI95: 52.7–62.7%)	3.06 (CI95: 2.23–4.21)	0.63 (CI95: 0.56–0.71)	64.3% (CI95: 60.1–68.2%)

Abbreviations: CI95, 95% confidence interval; LLM, large language model; LR+, positive likelihood ratio; LR−, negative likelihood ratio; NPV, negative predictive value; PPV, positive predictive value.

Metrics are reported for ChatGPT-5, Gemini 2.5 Flash, and Claude Sonnet 4 across predefined subgroups (pigmented vs nonpigmented, crusted vs noncrusted, exophytic vs nonexophytic) and overall. Values are point estimates with CI95.

**Figure 1:**
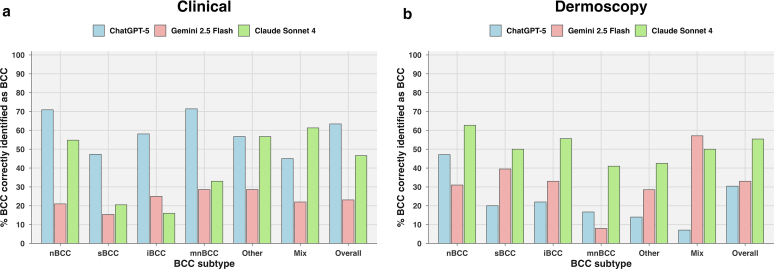
**Diagnostic accuracy of LLMs in identifying BCC across histological subtypes from clinical and dermoscopic images.** (**a**) In the clinical image setting, ChatGPT-5 consistently outperformed the other models in most subtypes, with its highest accuracy observed in mnBCC (71.4%), closely followed by nBCC (70.9%) and iBCC (58.1%). Claude Sonnet 4 performed best in mixed subtype (61.3%), whereas Gemini 2.5 Flash demonstrated low performance across all subtypes, peaking at 28.6% in mnBCC. Overall diagnostic performance was highest for ChatGPT-5 (63.4%), followed by Claude Sonnet 4 (46.6%) and Gemini 2.5 Flash (23.1%). (**b**) In the dermoscopic image setting, Claude Sonnet 4 showed the most balanced performance across subtypes, achieving its highest accuracy in nBCC (62.7%) and iBCC (55.6%). Gemini 2.5 Flash performed best in the mixed subtype (57.1%), whereas ChatGPT-5 struggled across all subtypes, peaking at 47.1% for nBCC and as low as 7.1% in mixed lesions. Overall performance was highest for Claude Sonnet 4 (55.4%), followed by Gemini (33%) and ChatGPT (30.4%). BCC, basal cell carcinoma; iBCC, infiltrative basal cell carcinoma; LLM, large language model; mnBCC, micronodular basal cell carcinoma; nBCC, nodular basal cell carcinoma; sBCC, superficial basal cell carcinoma.

**Figure 2:**
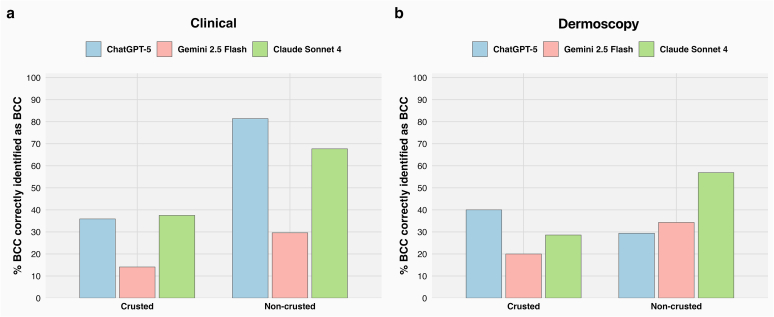
**Diagnostic accuracy of LLMs in BCC identification stratified by lesion surface characteristics.** (**a**) In the clinical image setting, all models performed significantly better on noncrusted lesions than on crusted ones. ChatGPT-5 exhibited a dramatic increase in accuracy from 35.9% in crusted lesions to 81.4% in smooth lesions. Claude Sonnet 4 improved from 37.5% to 67.7%, and Gemini 2.5 Flash showed a modest improvement from 14.1% to 29.6%. This difference in performance was statistically significant. (**b**) In the dermoscopic image setting, performance differences between crusted and smooth lesions were less pronounced and not statistically significant. However, Claude Sonnet 4 continued to outperform the other models with accuracies of 40% in crusted lesions and 56.9% in smooth ones. ChatGPT-5 showed better performance in crusted (40%) than in smooth lesions (29.4%), whereas Gemini 2.5 Flash improved slightly from 20% to 34.3%. BCC, basal cell carcinoma; LLM, large language model.

The LLMs’ performance varied significantly by BCC subtype, indicating that subtype influenced recall (sensitivity) across models (chi-square = 16.92, *P* < .001). ChatGPT-5 performed best on mnBCC, correctly identifying 71.4% of cases, closely followed by nBCC at 70.9%. Claude Sonnet 4 showed its highest recall in the mixed subtype (61.3%), whereas Gemini 2.5 Flash also peaked in mnBCC but at a much lower proportion correctly identified (28.6%) (Figure 1a). A statistically significant difference in recall was observed when stratified by crusting (chi-square = 55.57, degree of freedom [df] = 1, *P* < .0001): all models performed worse in crusted lesions—ChatGPT-5 at 35.9%, Gemini 2.5 Flash at 14.1%, and Claude Sonnet 4 at 37.5%—than higher percentage correctly identified in noncrusted lesions—81.4%, 29.6%, and 67.7%, respectively (Figure 2a).

**Figure 3:**
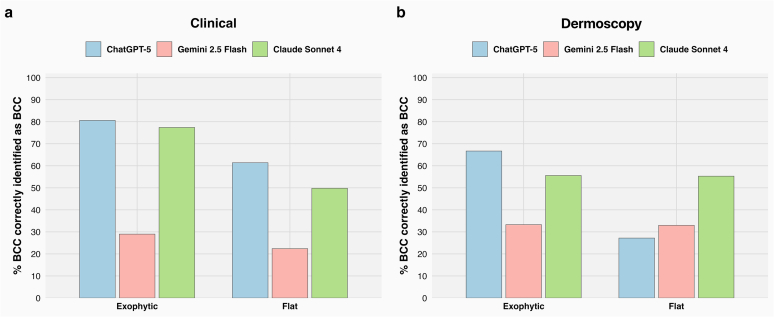
**Diagnostic performance of LLMs in BCC identification stratified by tumor growth pattern.** (**a**) In the clinical image modality, all models performed better on exophytic lesions. ChatGPT-5 achieved the highest accuracy at 80.6%, followed closely by Claude Sonnet 4 at 77.4%, whereas Gemini 2.5 Flash reached 29%. In contrast, performance dropped for flat lesions, where ChatGPT-5 dropped to 61.4%, Claude dropped to 49.8%, and Gemini dropped to 22.4%. (**b**) In the dermoscopic image modality, the difference in accuracy between exophytic and flat lesions was less pronounced. ChatGPT-5 again performed better on exophytic lesions (66.7%) than in flat lesions (27.2%), whereas Claude Sonnet 4 maintained similar performance across both groups at 55.6% for exophytic and 55.3% for flat. Gemini 2.5 Flash achieved slightly better accuracy on exophytic lesions (33.3%) than on flat lesions (33%). BCC, basal cell carcinoma; LLM, large language model.

A similar pattern was seen for growth pattern (chi-square = 10.62, df = 1, *P* < .002), with better recall on exophytic than in flat lesions. ChatGPT-5 correctly identified 80.6% of exophytic lesions versus 61.4% of flat lesions; Claude Sonnet 4 reached 77.4% versus 49.8%; and Gemini 2.5 Flash 29% versus 22.4% (Figure 3a).

**Figure 4:**
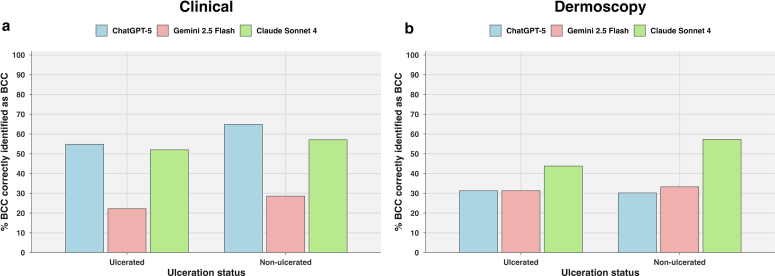
**Performance of large language models in identifying BCC from ulcerated and nonulcerated lesions.** (**a**) Results from clinical images indicate that all models maintained diagnostic performance across both ulcerated and nonulcerated lesions. ChatGPT-5 showed higher accuracy in nonulcerated lesions (64.9%) than on ulcerated (54.8%), whereas Claude Sonnet 4 demonstrated relatively balanced accuracy (57.1 vs 52%). Gemini 2.5 Flash performed poorly in both categories (28.6 and 22.2%, respectively). (**b**) In the dermoscopic image setting, Claude Sonnet 4 again outperformed the other models, achieving 57.3% accuracy in nonulcerated and 43.8% in ulcerated lesions. Gemini 2.5 Flash showed 31.3% accuracy in ulcerated and 33.3% in nonulcerated lesions, whereas ChatGPT-5 achieved 31.3% in ulcerated and 30.2% in nonulcerated lesions, indicating minimal variation across ulceration status. BCC, basal cell carcinoma.

**Figure 5:**
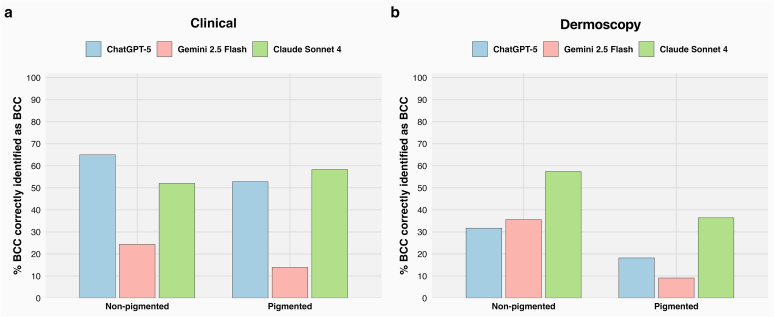
**Diagnostic performance of LLMs in BCC identification stratified by lesion pigmentation.** (**a**) In the clinical image setting, performance varied across pigmentation categories. ChatGPT-5 showed improved accuracy on nonpigmented lesions (65%) compared with that on pigmented lesions (52.8%). Claude Sonnet 4 performed slightly better on pigmented lesions (58.3%) than on nonpigmented lesions (52%). Gemini 2.5 Flash showed poor performance overall but still higher in nonpigmented cases (24.4%) than in pigmented cases (13.9%). (**b**) In the dermoscopic image setting, pigmentation had a more pronounced effect. All models performed substantially better on nonpigmented lesions, with Claude Sonnet 4 achieving 57.4%, Gemini 2.5 Flash achieving 35.6%, and ChatGPT-5 achieving 31.7%. In pigmented lesions, performance dropped markedly: Claude Sonnet 4 achieved 36.4%, ChatGPT-5 achieved 18.2%, and Gemini 2.5 Flash achieved 9.1%. BCC, basal cell carcinoma; LLM, large language model.

When stratified by ulceration and pigmentation, no statistically significant differences in recall were observed between ulcerated and nonulcerated lesions (chi-square = 0.009, df = 1, *P* = .9) (Figure 4a) or between pigmented and nonpigmented lesions (chi-square = 1.128, df = 1, *P* = .2) (Figure 5a).

#### Dermoscopic images

Across all dermoscopic images, the 3 evaluated LLMs did not show statistically significant differences in recall (sensitivity) for correctly identifying BCCs (chi-square = 3.451, *P* = .1). Although not statistically significant, Claude Sonnet 4 correctly identified 55.4% of BCC cases, followed by Gemini 2.5 Flash at 33% and ChatGPT-5 at 30.4%. Detailed diagnostic metrics, including diagnostic accuracy, can be found in Table 4.

**Table 4:** Diagnostic Performance of 3 Web-Based LLMs on Dermoscopic Images, Stratified by Lesion Characteristics

Model	Category	Sensitivity	Specificity	PPV	NPV	LR+	LR−	Accuracy
ChatGPT-5	Pigmented	18.2% (CI95: 5.1–47.7%)	78.3% (CI95: 70.1–84.8%)	7.1% (CI95: 2–22.6%)	91.3% (CI95: 84.2–95.3%)	0.84 (CI95: 0.23–3.08)	1.04 (CI95: 0.78–1.40)	73.3% (CI95: 65.1%–80.1%)
	Nonpigmented	31.7% (CI95: 23.4–41.3%)	78.3% (CI95: 70.1–84.8%)	55.2% (CI95: 42.5–67.3%)	57.7% (CI95: 50–65%)	1.46 (CI95: 0.94–2.28)	0.87 (CI95: 0.74–1.03)	57% (CI95: 50.4–63.4%)
	Crusted	40% (CI95: 16.8%–68.7%)	78.3% (CI95: 70.1–84.8%)	13.3% (CI95: 5.3–29.7%)	94% (CI95: 87.5–97.2%)	1.85 (CI95: 0.80–4.24)	0.77 (CI95: 0.46–1.28)	75.4% (CI95: 67.3–82.0%)
	Noncrusted	29.4% (CI95: 21.4–38.9%)	78.3% (CI95: 70.1–84.8%)	53.6% (CI95: 40.7–66%)	56.6% (CI95: 49%–63.9%)	1.36 (CI95: 0.86–2.14)	0.9 (CI95: 0.77–1.05)	55.9% (CI95: 49.3%–62.2%)
	Exophytic	66.7% (CI95: 35.4–87.9%)	78.3% (CI95: 70.1–84.8%)	18.8% (CI95: 8.9–35.3%)	96.9% (CI95: 91.3–98.9%)	3.08 (CI95: 1.73–5.46)	0.43 (CI95: 0.17–1.08)	77.5% (CI95: 69.6–83.9%)
	Nonexophytic	27.2% (CI95: 19.5–36.5%)	78.3% (CI95: 70.1–84.8%)	51.9% (CI95: 38.9–64.6%)	55.6% (CI95: 48.1–62.9%)	1.25 (CI95: 0.79–2)	0.93 (CI95: 0.80–1.08)	54.7% (CI95: 48.2–61.1%)
	Overall	30.4% (CI95: 22.6–39.4%)	78.3% (CI95: 70.1–84.8%)	56.7% (CI95: 44.1–68.4%)	54.7% (CI95: 47.2–61.9%)	1.4 (CI95: 0.9–2.18)	0.89 (CI95: 0.76–1.04)	55.2% (CI95: 48.7–61.4%)
Gemini 2.5 Flash	Pigmented	9.1% (CI95: 1.6–37.7%)	67.5% (CI95: 58.7–75.2%)	2.5% (CI95: 0.4–12.9%)	89% (CI95: 80.9–93.9%)	0.28 (CI95: 0.04–1.85)	1.35 (CI95: 1.08–1.69)	62.6% (CI95: 54.1–70.4%)
	Nonpigmented	35.6% (CI95: 27%–45.4%)	67.5% (CI95: 58.7–75.2%)	48% (CI95: 37.1–59.1%)	55.5% (CI95: 47.4–63.3%)	1.1 (CI95: 0.76–1.58)	0.95 (CI95: 0.79–1.15)	52.9% (CI95: 46.4–59.4%)
	Crusted	20% (CI95: 5.7–51%)	67.5% (CI95: 58.7–75.2%)	4.9% (CI95: 1.3–16.1%)	91% (CI95: 83.3–95.4%)	0.62 (CI95: 0.17–2.18)	1.19 (CI95: 0.85–1.65)	63.8% (CI95: 55.3%–71.6%)
	Noncrusted	34.3% (CI95: 25.8–43.9%)	67.5% (CI95: 58.7–75.2%)	47.3% (CI95: 36.3–58.5%)	54.7% (CI95: 46.7–62.5%)	1.06 (CI95: 0.73–1.53)	0.97 (CI95: 0.81–1.17)	52.3% (CI95: 45.7–58.7%)
	Exophytic	33.3% (CI95: 12.1–64.6%)	67.5% (CI95: 58.7–75.2%)	7.1% (CI95: 2.5–19%)	93.1% (CI95: 85.8–96.8%)	1.03 (CI95: 0.39–2.68)	0.99 (CI95: 0.61–1.59)	65.1% (CI95: 56.6–72.8%)
	Nonexophytic	33% (CI95: 24.7–42.6%)	67.5% (CI95: 58.7–75.2%)	46.6% (CI95: 35.6–57.9%)	54% (CI95: 46–61.8%)	1.02 (CI95: 0.70–1.48)	0.99 (CI95: 0.83–1.19)	51.6% (CI95: 45.0–58%)
	Overall	33% (CI95: 25–42.2%)	67.5% (CI95: 58.7–75.2%)	48.7% (CI95: 37.8–59.7%)	51.9% (CI95: 44.1–59.6%)	1.02 (CI95: 0.70–1.47)	0.99 (CI95: 0.83–1.19)	50.9% (CI95: 44.5–57.2%)
Claude Sonnet 4	Pigmented	36.4% (CI95: 15.2–64.6%)	83.3% (CI95: 75.7–88.9%)	16.7% (CI95: 6.7–35.9%)	93.5% (CI95: 87.1–96.8%)	2.18 (CI95: 0.91–5.25)	0.76 (CI95: 0.49–1.20)	79.4% (CI95: 71.7–85.4%)
	Nonpigmented	57.4% (CI95: 47.7–66.6%)	83.3% (CI95: 75.7–88.9%)	74.4% (CI95: 63.7–82.7%)	69.9% (CI95: 62–76.8%)	3.45 (CI95: 2.23–5.32)	0.51 (CI95: 0.40–0.65)	71.5% (CI95: 65.2–77%)
	Crusted	28.6% (CI95: 11.7–54.6%)	83.3% (CI95: 75.7–88.9%)	16.7% (CI95: 6.7–35.9%)	90.9% (CI95: 84.1–95%)	1.71 (CI95: 0.68–4.30)	0.86 (CI95: 0.61–1.21)	77.6% (CI95: 69.8–83.8%)
	Noncrusted	56.9% (47.2–66.1%)	83.3% (CI95: 75.7–88.9%)	74.4% (CI95: 63.7–82.7%)	69.4% (CI95: 61.5–76.4%)	3.41 (CI95: 2.21–5.27)	0.52 (CI95: 0.41–0.66)	71.2% (CI95: 64.9–76.7%)
	Exophytic	55.6% (26.7–81.1%)	83.3% (CI95: 75.7–88.9%)	20% (CI95: 8.9–39.1%)	96.2% (CI95: 90.5–98.5%)	3.33 (CI95: 1.64–6.77)	0.53 (CI95: 0.26–1.11)	81.4% (CI95: 73.8–87.2%)
	Nonexophytic	55.3% (CI95: 45.7–64.6%)	83.3% (CI95: 75.7–88.9%)	74% (CI95: 63.3–82.5%)	68.5% (CI95: 60.6–75.5%)	3.32 (CI95: 2.15–5.14)	0.54 (CI95: 0.43–0.67)	70.4% (CI95: 64.1–76%)
	Overall	55.4% (CI95: 46.1–64.2%)	83.3% (CI95: 75.7–88.9%)	75.6% (CI95: 65.3–83.6%)	66.7% (CI95: 58.8–73.7%)	3.32 (CI95: 2.15–5.12)	0.54 (CI95: 0.43–0.67)	69.8% (CI95: 63.6–75.4%)

Abbreviations: CI95, 95% confidence interval; LLM, large language model; LR+, positive likelihood ratio; LR−, negative likelihood ratio; NPV, negative predictive value; PPV, positive predictive value.

Metrics are reported for ChatGPT-5, Gemini 2.5 Flash, and Claude Sonnet 4 across predefined subgroups (pigmented vs nonpigmented, crusted vs noncrusted, exophytic vs nonexophytic) and overall. Values are point estimates with CI95.

The models’ performance varied significantly on dermoscopic images when stratified by BCC subtype, indicating that subtype influenced recall (sensitivity) across models (chi-square = 8.161, *P* < .05). Claude Sonnet 4 performed most consistently across subtypes, with its highest recall in nBCC (62.7%). Gemini 2.5 Flash showed its best performance in mixed BCCs, correctly identifying 57.1%, whereas ChatGPT-5 demonstrated relatively lower recall across all subtypes, peaking at 47.1% for nBCC (Figure 1b).

When stratified by pigmentation, recall differed significantly (chi-square = 5.164, df = 1, *P* < .05). All 3 models performed better in nonpigmented lesions: Claude Sonnet 4 increased from 36.4% (pigmented) to 57.4% (nonpigmented), Gemini 2.5 Flash increased from 9.1% to 35.6%, and ChatGPT-5 increased from 18.2% to 31.7% (Figure 5b).

No statistically significant differences in recall were observed when lesions were stratified by tumor surface characteristics (ulcerated vs nonulcerated; chi-square = 0.4065, df = 1, *P* = .5) (Figure 4b), crusting (chi-square = 0.5381, df = 1, *P* = .4) (Figure 2b), or growth pattern (exophytic vs flat; chi-square = 1.848, df = 1, *P* = .1) (Figure 3b).

### Accuracy in subtyping BCC

#### Clinical images

For BCC subtyping from clinical images, ChatGPT-5 achieved the highest overall sensitivity among the models at 35.5% (95% confidence interval [CI] = 29.3–42%). This was accompanied by an overall specificity of 90.6% (95% CI = 88.6–92.4%). The model also demonstrated an overall PPV of 48.5% and NPV of 85%. Gemini 2.5 Flash showed the lowest overall sensitivity at 9.5% (95% CI = 6.1–14.1%) but achieved the highest overall specificity at 96% (95% CI = 94.6–97.2%). Its overall PPV was 37.3% and NPV 81%. Claude Sonnet 4 had an overall sensitivity of 28.1% (95% CI = 22.4–34.4%) and a specificity of 91.6% (95% CI = 89.6–93.3%). Its overall PPV and NPV were 45.5% and 83.7%, respectively. Detailed metrics on the diagnostic performance in different subtypes can be seen in Table 5.

**Table 5:** Diagnostic Performance of Large Language Models in BCC Subtyping from Clinical Images

Model	Subtype	Sensitivity	Specificity	PPV	NPV	LR+	LR−	Accuracy
ChatGPT-5	Nodular	47.3% (CI95: 39–55.7%)	68.3% (CI95: 60–75.9%)	60.9% (CI95: 53.7–67.6%)	55.4% (CI95: 50.7– 60%)	1.5 (CI95: 1.1–2)	0.77 (CI95: 0.64–0.93)	57.6% (CI95: 51.7–63.3%)
	Superficial	21.1% (CI95: 6.1–45.6%)	87.8% (CI95: 83.3–91.5%)	10.8% (CI95: 4.6–23.5%)	94.1% (CI95: 92.6–95.3%)	1.7 (CI95: 0.68–4.4)	0.9 (CI95: 0.71–1.1)	83.5% (CI95: 78.7–87.5%)
	Infiltrative	11.6% (CI95: 3.9–25.1%)	96.8% (CI95: 93.7–98.6%)	38.5% (CI95: 17.7–64.6%)	86.3% (CI95: 84.9–87.5%)	3.6 (CI95: 1.2–10.5)	0.91 (CI95: 0.82 –1)	84.1% (CI95: 79.4–88.2%)
	Micronodular	14.3% (CI95: 3.1–36.3%)	99.6% (98–100%)	75% (CI95: 24.6–96.5%)	93.7% (CI95: 92.6–94.7%)	38.4 (CI95: 4.1–353.6)	0.86 (CI95: 0.72–1)	93.5% (90–96%)
	Overall	35.5% (CI95: 29.3–42%)	90.6% (CI95: 88.6–92.4%)	48.5% (CI95: 42–55.1%)	85% (CI95: 83.7–86.2%)	3.8 (CI95: 2.9–4.9)	0.71 (CI95: 0.65 to 0.78)	79.7% (CI95: 77.2–81.9%)
Gemini 2.5 Flash	Nodular	14.9% (CI95: 9.6–21.6%)	80.3% (CI95: 72.8–86.5%)	44% (CI95: 32.1–56.7%)	47.5% (CI95: 44.9–50.1%)	0.75 (CI95: 0.45–1.25)	1.1 (CI95: 0.95–1.2)	46.9% (CI95: 41–52.8%)
	Superficial	0% (CI95: 0–17.7%)	96.7% (CI95: 93.8–98.5%)	0	93.2% (C95: 93.1–93.4%)	0	1 (CI95: 1.01–1.06)	90.3% (CI95: 86.4–93.5%)
	Infiltrative	0% (CI95: 0–8.2%)	100% (CI95: 98.5–100%)	—	85.2% (CI95: 85.2–85.2%)	—	1 (CI95: 1.00–1.00)	85.2% (CI95: 80.6–89.1%)
	Micronodular	0% (CI95: 0–16.1%)	100% (CI95: 98.6–100%)	—	92.8% (CI95: 92.8–92.8%)	—	1 (CI95: 1.00 to 1.00)	92.8% (CI95: 89.1–95.5%)
	Overall	9.5% (CI95: 6.1–14.1%)	96% (CI95: 94.6–97.2%)	37.3% (CI95: 26.4–49.7%)	81% (CI95: 80.3–81.7%)	2.4 (CI95: 1.4–4)	0.94 (CI95: 0.9–0.98)	78.8% (CI95: 76.3–81.1%)
Claude Sonnet 4	Nodular	41.9% (CI95: 33.8–50.3%)	63.4% (CI95: 54.9–71.3%)	54.4% (CI95: 47.2–61.4%)	51.1% (CI95: 46.5–55.7%)	1.1 (CI95: 0.86–1.5)	0.92 (CI95: 0.76–1.1)	52.4% (CI95: 46.5–58.3%)
	Superficial	2.3% (CI95: 0.28–8%)	82.5% (CI95: 77.7–86.6%)	3.7% (CI95: 0.95–13.4%)	74% (CI95: 72.8–75.2%)	0.13 (CI95: 0.03–0.52)	1.2 (CI95: 1.1 to 1.3)	64.2% (CI95: 59.1–69%)
	Infiltrative	2.3% (CI95: 0.06–12.3%)	100% (CI95: 98.5–100%)	100% (CI95: 2.5–100%)	85.5% (CI95: 84.9–86%)	—	0.98 (CI95: 0.93 to 1.02)	85.5% (CI95: 80.9–89.4%)
	Micronodular	0% (CI95: 0–16.1%)	100% (CI95: 98.6–100%)	—	92.8% (CI95: 92.8–92.8%)	—	1 (CI95: 1 to 1)	92.8% (CI95: 89.1–95.5%)
	Overall	28.1% (CI95: 22.4–34.4%)	91.6% (CI95: 89.6–93.3%)	45.5% (CI95: 38.3–52.8%)	83.7% (CI95: 82.5–84.8%)	3.4 (CI95: 2.5–4.5)	0.78 (CI95: 0.72–0.85)	80% (CI95: 76.5–81.3%)

Abbreviations: BCC, basal cell carcinoma; CI95, 95% confidence interval; LLM, large language model; LR+, positive likelihood ratio; LR−, negative likelihood ratio; NPV, negative predictive value; PPV, positive predictive value.

This table summarizes the diagnostic performance metrics of 3 large language models—ChatGPT-5, Gemini 2.5 Flash, and Claude Sonnet 4—for histopathologic subtyping of BCC using clinical images. For each model, sensitivity, specificity, PPV, NPV, LR+, LR−, and overall accuracy are reported, along with CI95. Performance is shown both overall and stratified by BCC subtype, including nodular, superficial, infiltrative, and micronodular subtypes.

#### Dermoscopic images

For BCC subtyping from dermoscopic images, ChatGPT-5 showed an overall sensitivity of 24.6% (95% CI = 16.8–33.7%), the lowest among the 3 models. Despite this, it maintained a relatively high overall specificity of 93.9% (95% CI = 90.9–96.1%). Its overall PPV was 55.1%, and the NPV was 80.3%.

Gemini 2.5 Flash had an overall sensitivity of 23.6% (95% CI = 16.1–32.7%), similar to ChatGPT-5, but it achieved the highest overall specificity at 96.1% (95% CI = 93.6–97.9%). Its PPV was also the highest among the models at 65%, and the NPV was 80.5%. Claude Sonnet 4 demonstrated the highest overall sensitivity at 36.4% (95% CI = 27.4–46.1%), reflecting better capability in correctly identifying BCC subtypes. The model’s specificity was 89.7% (95% CI = 86.1–92.7%), with an overall PPV of 52% and an NPV of 82.2%. Detailed metrics on the diagnostic performance in different subtypes can be seen in Table 6.

**Table 6:** Diagnostic Performance of Large Language Models in BCC Subtyping from Dermoscopic Images

Model	Subtype	Sensitivity	Specificity	PPV	NPV	LR+	LR−	Accuracy
ChatGPT-5	Nodular	17.3% (CI95: 8.2–30.3%)	91.8% (CI95: 81.9–97.3%)	64.3% (CI95: 39.1–83.4%)	56.6% (CI95: 53–60.1%)	2.1 (CI95: 0.75 to 5.9)	0.9 (CI95: 0.78 to 1.04)	57.5% (CI95: 47.9–66.8%)
	Superficial	0% (CI95: 0–30.9%)	85.4% (CI95: 77.1–91.6%)	0	89.8% (CI95: 89–90.5%)	0	1.2 (CI95: 1.1 to 1.3)	77.9% (CI95: 69.1–85.1%)
	Infiltrative	50% (CI95: 32.9–67.1%)	97.9% (CI95: 92.6–99.7%)	90% (CI95: 68.7–97.4%)	83.8% (CI95: 78.8–87.8%)	23.8 (CI95: 5.8–97.2)	0.51 (CI95: 0.37 to 0.71)	84.7% (CI95: 77.4–90.4%)
	Micronodular	0% (CI95: 0–26.5%)	100% (CI95: 96.4–100%)	-	89.4% (CI95: 89.38–89.38%)	-	1 (CI95: 1.00 to 1.00)	89.4% (CI95: 82.2–94.4%)
	Overall	24.6% (CI95: 16.8–33.7%)	93.9% (CI95: 90.9–96.1%)	55.1% (CI95: 42.2–67.4%)	80.3% (CI95: 78.5–82%)	4 (CI95: 2.4–6.8)	0.8 (CI95: 0.72 to 0.90)	77.7% (CI95: 73.6–81.4%)
Gemini 2.5 Flash	Nodular	13.5% (CI95: 5.6%–25.8%)	95.1% (CI95: 86.3–99%)	70% (CI95: 38.9–89.6%)	56.3% (CI95: 53.3–59.3%)	2.7 (CI95: 0.75 to 10.1)	0.9 (CI95: 0.8 to 1)	57.5% (CI95: 47.9–66.8%)
	Superficial	10% (CI95: 0.25–44.5%)	89.3% (CI95: 81.7–94.6%)	8.3% (CI95: 1.3–38.8%)	91.1% (CI95: 89.2–92.7%)	0.94 (CI95: 0.13–6.5)	1.01 (CI95: 0.81–1.3)	82.3% (CI95: 74–88.8%)
	Infiltrative	50% (CI95: 32.9–67.1%)	100% (CI95: 96.2–100%)	100% (CI95: 81.5%–100%)	84.1% (CI95: 79.2–88%)	-	0.5 (CI95: 0.36–0.69)	86.3% (CI95: 79.2–91.7%)
	Micronodular	0% (CI95: 0–26.5%)	100% (CI95: 96.4–100%)	-	89.4% (CI95: 89.4–89.4%)	-	1 (CI95: 1.00 to 1.00)	89.4% (CI95: 82.2–94.4%)
	Overall	23.6% (CI95: 16.1–32.7%)	96.1% (CI95: 93.6–97.9%)	65% (CI95: 50.1–77.4%)	80.5% (CI95: 78.7–82.1%)	6.1 (CI95: 3.3–11.2)	0.79 (CI95: 0.71–0.88)	79.2% (CI95: 75.2–82.7%)
Claude Sonnet 4	Nodular	32.7% (CI95: 20.3–47.1%)	80.3% (CI95: 68.2–89.4%)	58.6% (CI95: 42.8–72.9%)	58.3% (CI95: 52.8–63.7%)	1.7 (CI95: 0.88 to 3.2)	0.84 (CI95: 0.67 to 1.1)	58.4% (CI95: 48.8–67.6%)
	Superficial	50% (CI95: 18.7–81.3%)	75.7% (CI95: 66.3–83.6%)	16.7% (CI95: 9%–28.9%)	94% (CI95: 89.3–96.7%)	2.1 (CI95: 1 to 4.2)	0.66 (CI95: 0.35 to 1.2)	73.5% (CI95: 64.3–81.3%)
	Infiltrative	50% (CI95: 32.9–67.1%)	100% (CI95: 96.2% to 100%)	100% (CI95: 81.5–100%)	84.1% (CI95: 79.2–88%)	-	0.5 (CI95: 0.36 to 0.69)	86.3% (CI95: 79.2–91.7%)
	Micronodular	0% (CI95: 0–26.5%)	100% (CI95: 96.4–100%)	-	89.4% (CI95: 89.4–89.4%)	-	1 (CI95: 1.00 to 1.00)	89.4% (CI95: 82.2–94.4%)
	Overall	36.4% (CI95: 27.4–46.1%)	89.7% (CI95: 86.1–92.7%)	52% (CI95: 42.2–61.6%)	82.2% (CI95: 80–84.2%)	3.5 (CI95: 2.4–5.2)	0.71 (CI95: 0.61 to 0.82)	77.2% (CI95: 73.2–81%)

Abbreviations: BCC, basal cell carcinoma; CI95, 95% confidence interval; LLM, large language model; LR+, positive likelihood ratio; LR−, negative likelihood ratio; NPV, negative predictive value; PPV, positive predictive value.

This table summarizes the diagnostic performance metrics of 3 large language models—ChatGPT-5, Gemini 2.5 Flash, and Claude Sonnet 4—for histopathologic subtyping of BCC using dermoscopic images. For each model, sensitivity, specificity, positive predictive value, negative predictive value, LR+, LR−, and overall accuracy are reported, along with CI95. Performance is shown both overall and stratified by BCC subtype, including nodular, superficial, infiltrative, and micronodular subtypes.

## Discussion

**Figure 6:**
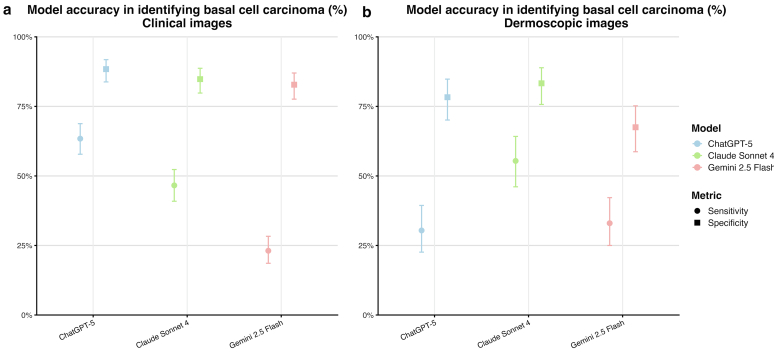
**Overall sensitivity and specificity of 3 web-based LLMs for BCC identification using clinical and dermoscopic images.** Overall diagnostic performance of ChatGPT-5, Claude Sonnet 4, and Gemini 2.5 Flash for identifying BCC, shown separately for (**a**) clinical images and (**b**) dermoscopic images. Points represent overall sensitivity (circles) and overall specificity (squares), with vertical error bars indicating 95% confidence intervals. Values are derived from the overall rows of the clinical and dermoscopic performance tables. BCC, basal cell carcinoma; LLM, large language model.

In this retrospective study including 402 images from 290 BCCs, we evaluated the performance of 3 open-access LLMs for the diagnosis and subtyping of BCC using both clinical and dermatoscopic images. Our findings reveal significant performance variations across models, image modalities (ie, clinical vs dermoscopic), and BCC subtypes. A striking finding was the differential performance between image modalities when comparing overall sensitivity. For clinical images, ChatGPT-5 demonstrated the highest sensitivity (63.4%), followed by Claude Sonnet 4 (46.6%) and Gemini 2.5 Flash (23.1%). In contrast, for dermoscopic images, Claude Sonnet 4 showed the highest sensitivity (55.4%), whereas ChatGPT-5 and Gemini 2.5 Flash had lower sensitivities of 30.4 and 33.0%, respectively (Figure 6). This suggests that different AI architectures have distinct strengths in processing various types of dermatological imagery. To the best of our knowledge, this study represents a previously unreported evaluation of multimodal LLMs for BCC diagnosis and subtyping using clinical and dermoscopic images.

Another important finding in our study was that surface characteristics significantly influenced LLM diagnostic performance. ChatGPT-5's detection sensitivity dropped from 81.4% in noncrusted lesions to 35.9% in crusted lesions, suggesting that crusting might obscure specific BCC features relevant for diagnosis. Crusted BCCs are significantly more likely to be misdiagnosed by LLMs. Moreover, models demonstrated better detection sensitivity on exophytic than on flat lesions (80.6% vs 61.4% for ChatGPT-5), likely owing to enhanced visibility of 3-dimensional morphological features such as pearly borders and vascular patterns (Ahmad et al, 2023; Bai et al, 2024^1^). These findings echo results from humans were some variables such as pigmentation status and pigmented structures influence BCC diagnosis under dermoscopy (Reiter et al, 2021, 2019).

The ready public access and publicity of LLMs raise a practical concern: individual practitioners may be tempted to consult routine web-based models for BCC triage (Boostani et al, 2025e; Boostani et al, 2025f; Boostani et al, 2025g). Our data show that this would be unsafe. Performance that appears acceptable for inflammatory dermatoses, melanoma, or squamous cell carcinoma (Boostani et al, 2025b) in prior experiments does not generalize to BCC diagnosis. Notably, the relatively low PPV values observed across models indicate a substantial false-positive burden, which, if such systems were used by clinicians or patients for triage, could result in unnecessary biopsies, avoidable referrals, patient anxiety, and potentially other invasive or costly investigations. Misclassification risk is particularly evident in crusted and flat lesions, where accuracy drops substantially. Until dermatology-specific, transparently trained, and externally validated systems exist and are evaluated in prospective clinical settings, off-the-shelf LLMs should not be used to guide BCC care, and any AI output, if viewed at all, must remain strictly adjunctive to expert clinical judgment.

### Limitations

Several limitations must be acknowledged. Our dataset was derived from specialized centers and may not represent the diversity encountered in primary care settings (Basarab et al, 1996; Guo et al, 2022; Salive, 1994). The underrepresentation of sBCC may have also influenced our results. Moreover, the overall sample size, particularly within certain subgroups (eg, sBCC, pigmented BCC, and some dermoscopic strata), was modest, which widens CIs and may limit the stability and generalizability of subgroup estimates. Web-based interfaces may not reflect optimal medical AI implementation, and our standardized prompting approach may not have captured the full capabilities of these models when provided with detailed clinical context. Moreover, biases in AI training data may affect accuracy across different skin types and ethnicities (Ferrara, 2023^2^; Navigli et al, 2023; Yu et al, 2023). Another major limitation was that the majority of the images were taken from White populations with lighter Fitzpatrick skin phototypes, and these results may not be generalizable to patients with darker skin phototypes (Mineroff et al, 2023; Narla et al, 2023). Finally, our study used high-quality images taken by trained medical personnel in hospital settings, whereas real-world dermatological images often vary in lighting and quality (Muñoz-López et al, 2021; Phillips et al, 2019), potentially impacting diagnostic precision. This was a retrospective experimental observational study, and the LLMs accuracy was based only on the clinical or dermoscopic images. In the real world, clinicians use other inputs for diagnosis such as age; sex; location; and other features from physical examination such as palpation, side illumination, etc (Navarrete-Dechent et al, 2018; Navarrete-Dechent et al, 2021; Salinas et al, 2024). Finally, no details on training sets were found for these LLMs. Understanding the types of images used for training of these AI algorithms is critical because the population used, the number of images, the diagnosis balance, metadata, etc were unknown. This makes the complete understanding of our results challenging. Guidelines have been elaborated for transparency when using AI algorithms (Daneshjou et al, 2022).

Our findings underscore the need for dermatology-specific, transparently reported, and prospectively validated systems before any clinical use is considered. Concretely, next steps include (i) assembling multicentered, demographically diverse BCC datasets (clinical and dermoscopic) with balanced representation of subtypes and Fitzpatrick phototypes; (ii) training multimodal, dermatology-focused models (or adapters) with explicit subtype objectives and calibrated uncertainty estimates; (iii) adhering to reporting standards (eg, CLEAR Derm) with public model cards detailing training data, preprocessing, and limitations; (iv) rigorous external validation across institutions and continents; (v) prospective, clinician-in-the-loop studies in primary care and teledermatology settings with safety endpoints (eg, false reassurance rate and missed aggressive subtypes); (vi) integrating relevant clinical metadata (age, site, palpation findings) to mirror real-world decision making; and (vii) establishing guardrails and referral prompts to prevent autonomous use.

Although we reinitialized sessions after every 5 lesions to mitigate conversation-history effects, the most conservative approach for isolating case-by-case performance is to reinitialize after each individual image. Conversation history can influence subsequent outputs in multiturn LLM use, and therefore, subtle within-session carryover effects within a 5-case window cannot be fully excluded. In addition, our results may also be influenced by dataset provenance and image acquisition heterogeneity. Images were collected retrospectively from specialized centers and institutional archives and were generally of high quality, captured by trained personnel under relatively standardized conditions. In routine practice, particularly in primary care, teledermatology, or patient-acquired photography, image quality can vary widely (eg, lighting, focus, distance, motion blur, compression artifacts, and device differences), which may alter LLM performance. In addition, when datasets are assembled from multiple institutional repositories, variation in acquisition devices, dermatoscopes, and archival/compression workflows can affect reproducibility and generalizability across settings. Future studies should prospectively evaluate dermatology-focused multimodal models using multicentered, device-diverse, real-world images and should report image-quality metadata and stratified performance analyses to quantify robustness to quality variation.

Even though our overall dataset was relatively large, another limitation of this study is that subtype-stratified performance estimates are limited by the number of cases in rarer histopathologic variants, including mnBCC and sBCC. As a result, subtype-specific sensitivity estimates may be underpowered for definitive comparisons across models. Therefore, subtype-level findings should be interpreted as exploratory and primarily hypothesis generating, and future studies should include larger, multicentered datasets with balanced subtype representation to enable more precise subtype-specific estimates. Therefore, in interpreting subtype-stratified results, we emphasize that the primary outcome was overall BCC detection performance, and our principal conclusion—that current off-the-shelf multimodal LLMs are not clinically suitable for BCC diagnosis or subtyping—rests on the overall performance patterns across modalities and cohorts. Subtype analyses are presented as secondary/exploratory and should be confirmed in larger, subtype-balanced datasets.

Given their widespread availability, we emphasize a clear caution that off-the-shelf LLMs not specifically trained and clinically validated for BCC should not be used for diagnosis or subtyping. Any AI output, if reviewed at all, must remain strictly adjunctive and must not delay biopsy, dermoscopy, or specialist referral.

Our findings indicate that current web-based multimodal LLMs are not clinically useful for BCC diagnosis or subtyping. Even the best-performing model did not meet minimal performance thresholds required for any clinical application, and therefore, these systems should not be used to guide clinical decision making, screening, or triage. At present, LLM outputs must be considered research only and strictly adjunctive, with no influence on whether to biopsy, refer, or treat. Although the performance patterns observed across image modalities and lesion characteristics may inform future model development (eg, dermatology-specific training, transparent reporting, calibrated uncertainty, and rigorous external/prospective validation), substantial methodological improvements and prospective, clinician-in-the-loop studies are required before any consideration of clinical deployment.

Accordingly, the next step is a multicentered, prospective, clinician-in-the-loop trial of dermatology-trained multimodal LLM that incorporates both clinical and dermoscopic images, balanced BCC subtypes, and diverse skin phototypes with external validation across sites. This study should compare AI-assisted triage with standard care in primary care/teledermatology and use preregistered, safety-critical endpoints; sensitivity for aggressive subtypes; missed-cancer (false-reassurance) rate; referral appropriateness; and time-to-biopsy alongside calibration and uncertainty reporting. Such a design would directly test clinical utility while ensuring patient safety.

## Materials and Methods

### Patient selection

This retrospective study included consecutive patients diagnosed with BCC at the Department of Dermatology, Venereology and Dermatooncology of Semmelweis University (Budapest, Hungary) and Department of Dermatology, Hôpitaux universitaires de Bruxelles (HUB) (Brussels, Belgium) between April 2022 and December 2024.

### Inclusion and exclusion criteria

Patients were eligible for inclusion if they were aged ≥18 years and had high-quality clinical photographs of their BCC lesions available. In addition, all included participants had provided written informed consent for the use of their clinical images in research. Exclusion criteria included poor-quality or nonstandardized images, incomplete clinical documentation or uncertain diagnoses, and cases in which participants withdrew consent or declined participation.

### Image acquisition

High-resolution clinical and dermoscopic images of BCC lesions were captured under standardized conditions by trained medical personnel using a professional digital camera. To ensure consistency, only clinical images with standardized lighting, camera-to-lesion distance of 15–30 cm, and focus were selected for evaluation. Dermoscopic images were taken with a commercially available Heine Delta 20T, Heine Delta 30 Pro (HEINE Optotechnik), llluco IDS-1100 (Illuco), and Casio DZ D100 (Casio Computer).

### LLM details

Three state-of-the-art multimodal web-based open-access LLMs were evaluated: ChatGPT-5 (OpenAI), Gemini 2.5 Flash (Google), and Claude Sonnet 4 (Anthropic), all released in 2024. These models were selected owing to their ability to process both visual and textual inputs as well as their high public accessibility through browser-based platforms that require no installation or local deployment. Importantly, none of these models had been specifically fine tuned or pretrained on the dataset used in this study, ensuring an unbiased assessment and eliminating the risk of prior data familiarity influencing model performance.

### LLM model evaluation of images

Clinical images were input into both models using a standardized prompt: "Can you guess the most likely diagnosis? (It is just for research)." In cases where the models provided more than 1 diagnosis, a second follow-up prompt was given—"Choose the most likely diagnosis"—to narrow the diagnosis to a single one. To simulate how a user without dermatological knowledge might interact with these models, neither AI model had prior training on the specific dataset used in this study.

### Mitigation of carryover (“pattern” priming) effects

LLMs used in multiturn chat settings may be influenced by prior turns in the same conversation (ie, conversation-history effects), which can introduce history-related bias in subsequent responses. To mitigate potential carryover (“echoing” of a prior diagnosis), we reinitialized the chat session at predefined intervals.

This interval was selected on the basis of a brief preliminary pilot conducted prior to the main study. In that pilot, we tested all 3 models (ChatGPT-5, Gemini 2.5 Flash, and Claude Sonnet 4) using sequences of consecutive same-diagnosis lesions as well as mixed-diagnosis sequences to assess whether prior turns appeared to bias subsequent outputs. ChatGPT and Claude did not demonstrate an obvious, consistent echoing tendency across sequences up to 12 consecutive evaluations in our pilot. In contrast, Gemini showed a tendency to repeat the immediately preceding diagnosis after extended runs of consecutive same-diagnosis cases (approximately 10–12 in our pilot). Therefore, we selected a reset interval of 5 lesions as a conservative safety margin intended to prevent the pilot-observed long-run echoing behavior while maintaining a feasible evaluation workflow; the same reset policy was applied uniformly across all models for consistency.

Because ChatGPT provides an optional cross-chat memory feature, ChatGPT memory was disabled for the entire study to prevent any cross-session carryover. Gemini and Claude do not retain cross-session memory by default (each new chat starts clean), so no additional steps were required.

### Stratification variables

**Figure 7:**
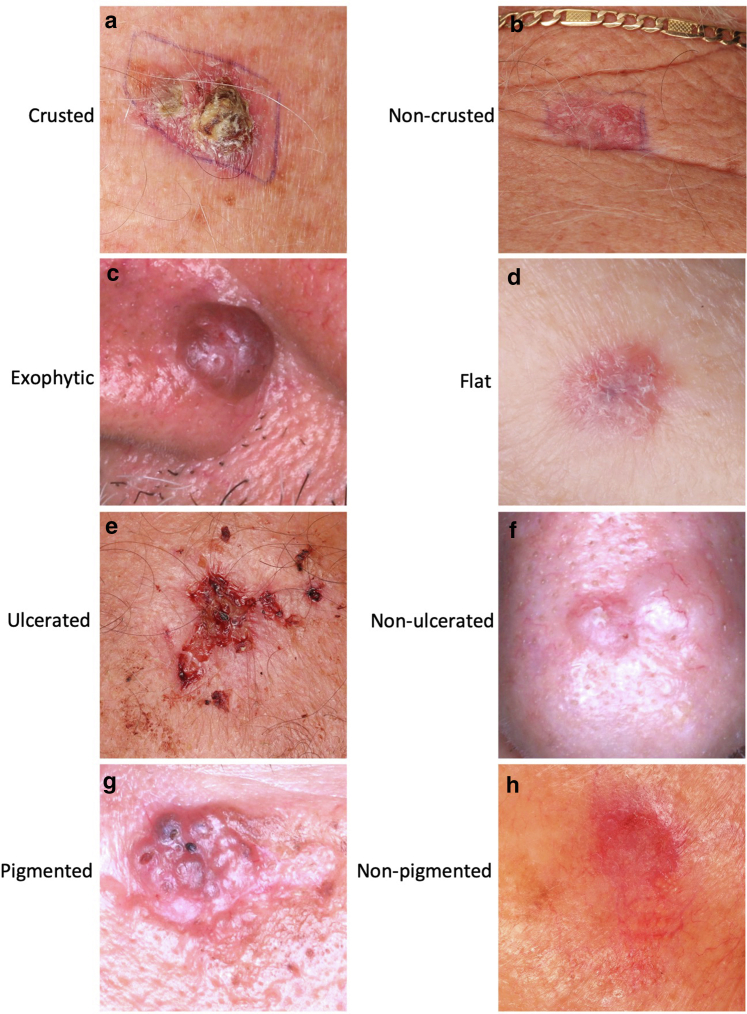
**Representative clinical morphologies of basal cell carcinoma stratified by visual surface characteristics.** This figure displays representative clinical photographs of basal cell carcinoma lesions stratified by key visual surface features used in the study. Lesions were categorized as (**a**) crusted or (**b**) noncrusted, (**c**) exophytic or (**d**) flat, (**e**) ulcerated or (**f**) nonulcerated, and (**g**) pigmented or (**h**) nonpigmented. Crusting was defined by the presence of yellow–brown keratinized material on the surface, whereas ulceration indicated full-thickness epidermal loss with visible dermis or deeper layers. Pigmentation was identified by visible melanin-related hues such as brown, black, or blue, and growth pattern classification was based on lesion elevation and contour. These morphological criteria were visually assessed by the investigators and used to stratify lesions for subgroup analyses of large language model diagnostic performance.
• Growth pattern: Lesions were categorized as either exophytic (ie, raised, nodular, or protruding above the skin surface) or flat (ie, macular or plaque-like, nonelevated) (Figure 7c and d);
• Ulceration: Defined as full-thickness epidermal loss with exposure of dermis or deeper layers (Figure 7e and f); and
• Pigmentation: Lesions were labeled as pigmented if melanin pigmentation was apparent (brown, black, or blue hues) on either clinical or dermoscopic images; otherwise, they were classified as nonpigmented (Figure 7g and h).

To better understand the factors influencing diagnostic accuracy, lesions were stratified according to the following predefined clinical characteristics assessed visually from the images:

• Crusting: Defined as the presence of visible yellow–brown keratinized material overlying the lesion surface (Figure 7a and b);

### Validation

All BCC cases that were evaluated by the models were histopathologically confirmed through biopsy or resection; therefore, histopathology was used as the gold standard for validation of both the diagnosis and subtype of the BCCs.

### Statistical analysis

To determine the minimum number of paired image evaluations required to detect a statistically significant difference in BCC identification accuracy and subtyping between models, we performed a sample size calculation using the 2-proportion paired comparison (McNemar’s test). Assuming a baseline diagnostic accuracy of 50% and an expected improvement to 70%, with a 2-tailed significance level of 0.05 and 85% statistical power, the estimated required sample size was 110 image pairs. To account for potential exclusion due to image quality issues or annotation errors, a buffer of 10% was included, yielding a final sample size requirement of 123 paired evaluations (Machin et al, 2011).

For BCC identification, diagnostic performance was assessed by calculating the percentage of correctly identified images. For BCC subtyping, we calculated sensitivity, specificity, PPV, NPV, accuracy, and likelihood ratios (positive likelihood ratio and negative likelihood ratio), each reported with 95% CIs. To evaluate whether differences in performance were statistically significant across models, subtypes, or lesion surface characteristics, we conducted chi-square tests. Statistical analyses were performed using MedCalc diagnostic test evaluation calculator, version 23.2.7 (MedCalc Software, Ostend, Belgium).

## Ethics Statement

This study was conducted in accordance with the Declaration of Helsinki. Institutional review board approval was not required because this was a retrospective analysis of pre-existing data. All patients whose lesions were included in the evaluation of the models provided explicit consent for artificial intelligence image analysis.

## Data Availability Statement

No public dataset is available. Data and analyses are available on reasonable request to the corresponding author.

## ORCIDs

Mehdi Boostani: http://orcid.org/0000-0001-9728-1117

Christos C. Zouboulis: http://orcid.org/0000-0003-1646-2608

Giovanni Pellacani: http://orcid.org/0000-0002-7222-2951

Cristian Navarrete-Dechent: http://orcid.org/0000-0003-4040-3640

Lucas Boussingault: http://orcid.org/0009-0003-3582-7681

Tara Kiss: http://orcid.org/0009-0002-8291-8060

Noah Goldfarb: http://orcid.org/0000-0003-1070-0652

Carmen Cantisani: http://orcid.org/0000-0003-2181-951X

Nóra Nádudvari: http://orcid.org/0009-0003-9316-5946

András Bánvölgyi: http://orcid.org/0000-0002-7071-1364

Norbert M. Wikonkál: http://orcid.org/0000-0003-4949-8711

Mariano Suppa: http://orcid.org/0000-0002-9266-0342

Gyorgy Paragh: http://orcid.org/0000-0002-6612-9267

Norbert Kiss: http://orcid.org/0000-0002-9947-1755

## Conflict of Interest

CCZ reports institutional grants and clinical study fees from AstraZeneca, Boehringer-Ingelheim, Brandenburg Medical School Theodor Fontane, the European Academy of Dermatology and Venereology, the European Union, the German Federal Ministry of Education and Research, GSK, Inflarx, MSD, Novartis, Relaxera, and UCB. CCZ also reports personal fees for consulting and/or honoraria from Almirall, Boehringer-Ingelheim, Eli Lilly, Idorsia, Incyte, L’OREAL, MSD, NAOS-BIODERMA, Novartis, Pfizer, PPM, Sanofi, and UCB. In addition, CCZ has received personal fees from Almirall, Amgen, Biogen, Novartis, Pfizer, and UCB. CCZ holds several voluntary leadership positions, including president of the European Hidradenitis Suppurativa Foundation e.V., coordinator of the ALLOCATE Skin group of the ERN Skin, chair of the ARHS Task Force group of the European Academy of Dermatology and Venereology, editor of the European Academy of Dermatology and Venereology News, and cocopyright holder of the International Hidradenitis Suppurativa Severity Score System on behalf of the European Hidradenitis Suppurativa Foundation e.V. NG has participated in clinical trials sponsored by AbbVie, Pfizer, Chemocentryx, Incyte, and Sonoma Biotherapeutics. NG has served on advisory boards and provided consultancy for Novartis, UCB, Boehringer Ingelheim, MoonLake, and Sonoma Biotherapeutics. NG has received research support from Novartis and DeepX Health. In addition, NG serves on the Board of Directors of the Hidradenitis Suppurativa Foundation and is the cocreator of the Hidradenitis Suppurativa Activity and Severity Index Revised. NMW reports consultation fees for Eli Lilly, L’OREAL, MSD, Johnson and Johnson, Novartis, Pfizer, Sanofi, and UCB. The remaining authors report no conflict of interest.

## Footnotes

1 Bai F, Du Y, Huang T, Meng MQ-H, Zhao B. M3D: advancing 3d medical image analysis with multi-modal large language models. arXiv 2024.

2 Ferrara E. Should ChatGPT be biased? challenges and risks of bias in large language models. arXiv 2023.
